# The Effect of Correlated Neuronal Firing and Neuronal Heterogeneity on Population Coding Accuracy in Guinea Pig Inferior Colliculus

**DOI:** 10.1371/journal.pone.0081660

**Published:** 2013-12-16

**Authors:** Oran Zohar, Trevor M. Shackleton, Alan R. Palmer, Maoz Shamir

**Affiliations:** 1 Deptartment of Physiol, Faculty of Health Sciences, Ben-Gurion University of the Negev, Be'er-Sheva, Israel; 2 MRC Institute of Hearing Research, University Park, Nottingham, United Kingdom; 3 Department of Physics, Faculty of Natural Sciences, Ben-Gurion University of the Negev, Be'er-Sheva, Israel; Instituto de Neurociencias de Alicante UMH-CSIC, Spain

## Abstract

It has been suggested that the considerable noise in single-cell responses to a stimulus can be overcome by pooling information from a large population. Theoretical studies indicated that correlations in trial-to-trial fluctuations in the responses of different neurons may limit the improvement due to pooling. Subsequent theoretical studies have suggested that inherent neuronal diversity, i.e., the heterogeneity of tuning curves and other response properties of neurons preferentially tuned to the same stimulus, can provide a means to overcome this limit. Here we study the effect of spike-count correlations and the inherent neuronal heterogeneity on the ability to extract information from large neural populations. We use electrophysiological data from the guinea pig Inferior-Colliculus to capture inherent neuronal heterogeneity and single cell statistics, and introduce response correlations artificially. To this end, we generate pseudo-population responses, based on single-cell recording of neurons responding to auditory stimuli with varying binaural correlations. Typically, when pseudo-populations are generated from single cell data, the responses within the population are statistically independent. As a result, the information content of the population will increase indefinitely with its size. In contrast, here we apply a simple algorithm that enables us to generate pseudo-population responses with variable spike-count correlations. This enables us to study the effect of neuronal correlations on the accuracy of conventional rate codes. We show that in a homogenous population, in the presence of even low-level correlations, information content is bounded. In contrast, utilizing a simple linear readout, that takes into account the natural heterogeneity, even of neurons preferentially tuned to the same stimulus, within the neural population, one can overcome the correlated noise and obtain a readout whose accuracy grows linearly with the size of the population.

## Introduction

In mammals, at low frequencies, sound source localization in the horizontal plane relies on interaural time differences [1]. Some models suggest that the first stages of the computation of interaural time differences are based on the detection of binaural correlations (BC) and tuning to BC has been observed in the auditory system [2]–[4].

Evidence from many brain regions, including the auditory system and in particular the inferior colliculus, show that the trial- to-trial fluctuations in the responses of different neurons are correlated [5]–[9]. Noise-correlations have been reported to be positively biased, although some debate still remains [10].Theoretical studies revealed that these correlations can have a considerably detrimental effect on the amount of information that can be encoded by the population response [11]–[13]. It is common to distinguish two types of correlations [7], [14]. One is the “signal correlations” that measures the correlation between the *mean* responses of different cells. For example, two cells with very similar tuning curves will show high signal correlations. Thus, the distribution of signal correlations can measure the heterogeneity within the neural population. The second is termed the “spike-count correlations” and measures the correlation between the fluctuations from the mean responses across different trials. The specific nature of interaction between these two types of correlations can lead to different results in terms of information content of the neural response [11], [13], [15]–[20]. Empirical studies report that noise-correlations also have a functionally dependent component, i.e., neurons with higher signal-correlations tend to show higher noise-correlation [8], [9], [21], [22] (however, the indiscriminant correlation is typically larger than the functional dependence). Theory has shown that the functional dependence of noise-correlations can have a major detrimental effect on the information capacity of the neural population [23].

Previously, Shamir and Sompolinsky [18] pointed out that the inherent neuronal heterogeneity may provide means to overcome the correlated noise. They studied the Fisher information and provided a perturbation-approach analysis that yielded a bound on the accuracy of a simple linear estimator, using a framework of a hypothesized statistical model for the neural responses. More recently Ecker and colleagues [20] provided an extended analysis of the Fisher information of a heterogeneous neural population, also in a modeling study. However, it is not clear how real neural heterogeneity will affect a simple biologically plausible readout mechanism such as a linear estimator, as both the perturbation approach and the Fisher information have been shown to yield a very poor estimate of such a readout [18], [20].

Here we use single cell recordings from the inferior colliculus (IC) of the guinea pig in response to different BC levels [2], to evaluate the effect of correlations and heterogeneity on the accuracy of a linear estimator. The utility of studying a linear estimator is twofold. One, it is generally assumed that a similar readout can be implemented by the central nervous system. Two, it provides a clear notion of signal and noise which enable a better understanding of the effects of correlations and heterogeneity. As the single cell recordings are not simultaneous, we cannot asses the true correlations. To study the effect of correlations based on single cell recordings, we employ an algorithm that generates a pseudo-population response with varying levels of correlations and heterogeneity, while preserving the marginal distribution of the cells and the inherent heterogeneity.

The outline of the paper is as follows. First we define our algorithm and apply it to study coding accuracy in homogeneous populations. Then we turn to investigate the case of a heterogeneous population. The homogeneous population is comprised of neurons which are identical in every way (they are, in fact responses from the same recorded neuron). The heterogeneous population is formed from all the neurons in our data set. Finally, we summarize our results and discuss the generality of our findings. The data used here were described in detail by Shackleton and colleagues [2], [24], [25] and their information theoretical properties were analyzed by Gordon et al. [26].

## Results

**Figure 1:**
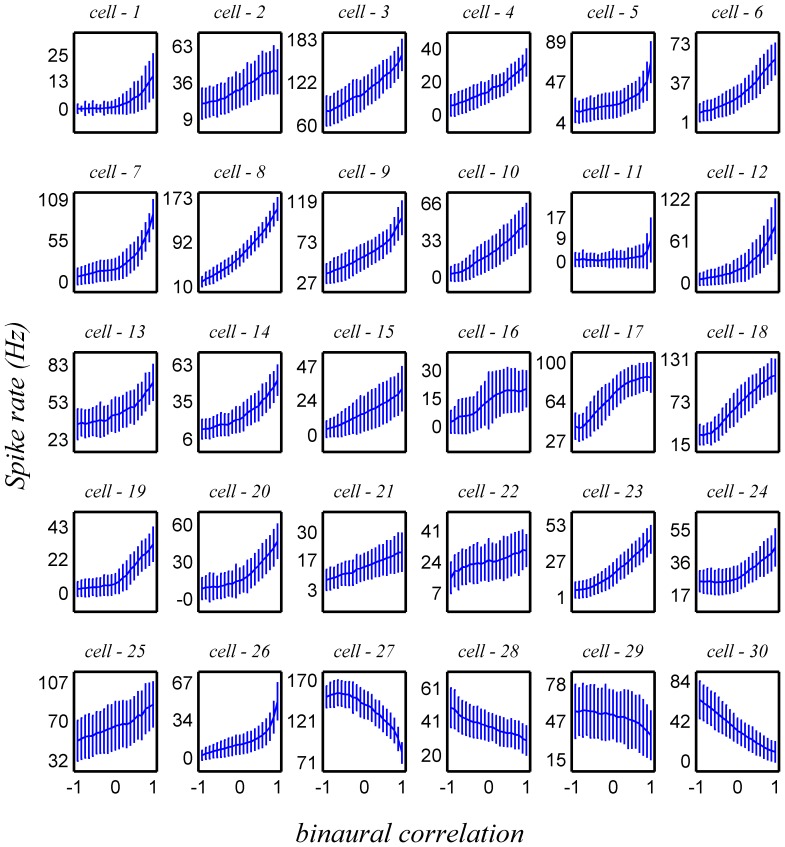
Neuronal heterogeneity. The conditional mean (over 200–500 trials for given stimulus BC) firing rate is plotted as a function of the binaural correlation level for the 30 different cells in the data set. The error bars depict the *standard deviation* of the firing rate. The firing rates were computed for the period of 100 ms following stimulus onset. Typically, the tuning curves are monotonic in the BC and approximately linear.

**Figure 2:**
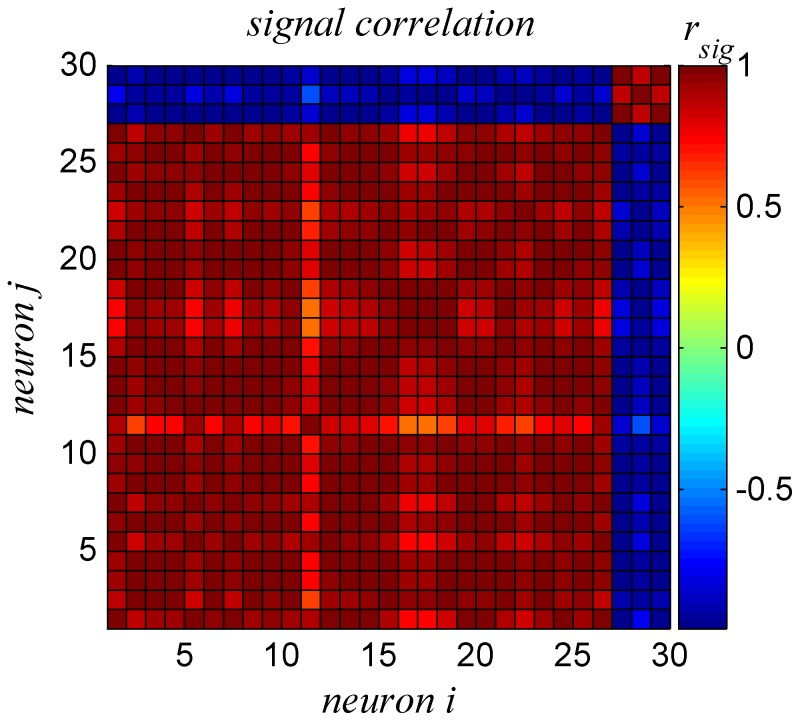
Signal correlations. The signal correlations, i.e., the Pearson correlations between the tuning curves of the different neurons, are shown in color code.

Figure 1 shows tuning curves of all the cells within the analyzed data set (mean ±std). Typically, the mean firing rate, *r*, is a monotonic function of BC and can be approximated by a linear function: 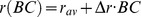, where 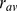 is the average firing rate across trials and across different stimulus conditions, and 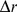 represents the modulation amplitude of the tuning curve with BC. Although qualitatively the tuning curves of many IC neurons are similar, quantitatively they are different. This inherent diversity, can be characterized by the distribution of the parameters 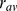 and 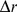. For example most cells are characterized by tuning curves with positive slopes; however about 15% of the cells (4 cells out of 30) have tuning curves with negative slopes. Alternatively, one can characterize the diversity by the signal correlations, which are the Pearson correlations between the tuning curves of the different neurons. Figure 2 shows the signal correlations within the different neurons in our dataset. As can be seen from the Figure, the dataset is composed of two subpopulations: one with positive slope tuning curves and the other with negative slope. Within each subpopulation the signal correlations are very high, and are close to one for the population with positive slopes. On the other hand, signal correlations between the two populations are very negative.

Signal correlations measure the similarity of the *mean* response. However, the neural responses fluctuate around their mean from trial to trial. To distinguish between the BC, that characterize the physical stimulus, and the correlations between the fluctuations of the neural responses we shall term the neural response correlations ‘spike-count correlations’. We shall refer to the correlations of the auditory stimulus between the two ears only by the term BC (or model parameter *θ*). The spike-count fluctuations and their correlations limit the amount of information that can be extracted from the neural responses. Here, we study the effect of spike-count correlations on the ability to accumulate information from large populations of neurons. Below, we will first demonstrate the strong detrimental effect of spike-count correlations on the information content of a homogeneous population. Then, we will analyze the information content of the neural responses in a heterogeneous population and show that, in this case, information content is not limited by the spike-count correlations.

### Correlations in an homogenous population

**Figure 3:**
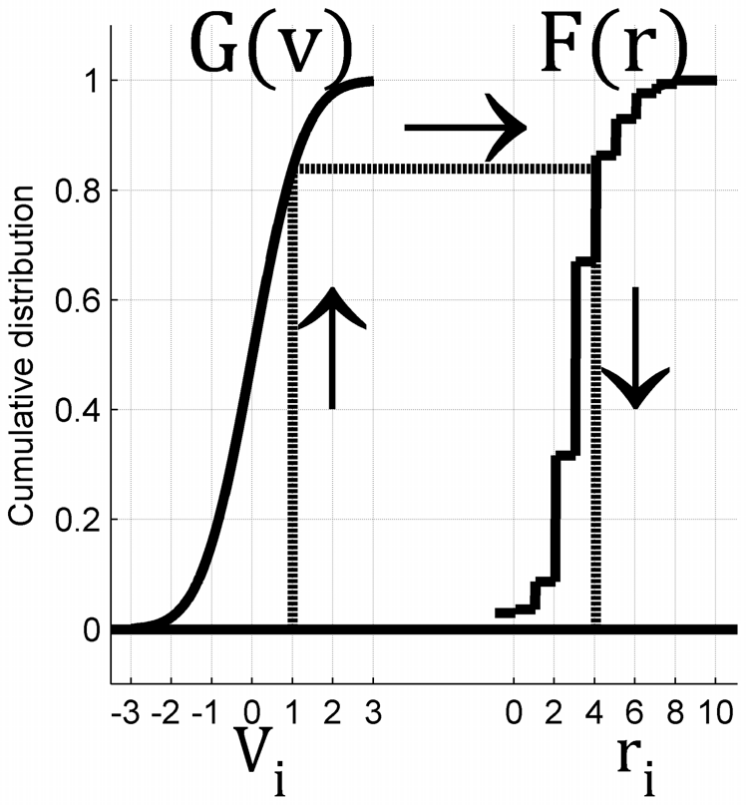
Illustration of Step 2 of our algorithm: translating the continuous input variable to the discrete neural response. The figure depicts the two cumulative distribution functions, 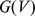 [left, cumulative distribution of Gaussian input variable] and 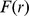 [right, empirical cumulative distribution of neuronal spike-count]. In the specific example shown in the figure, the random input variable is 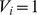. The cumulative distribution of having input of equal or less than 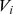 is in this case 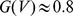. To translate the input variable to spike count we choose the number of spikes that corresponds to the same cumulative distribution. In the specific example of the illustration, this corresponds to firing of four spikes, 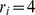.

**Figure 4:**
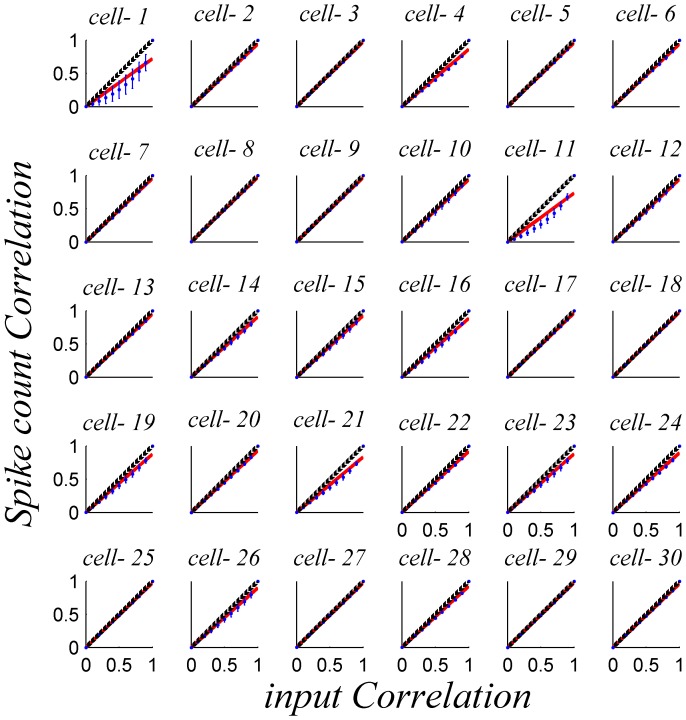
Mean spike-count correlation coefficient 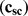 as function of input correlation 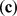 for homogeneous pseudo-populations. Pseudo-population responses of 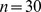 neurons were generated based on the response distribution of each of the 30 neurons in our data set, shown by the different panels. To compute the spike count correlations we simulated the response of the pseudo population for 1000 trials for every stimulus condition. The correlation coefficient matrix was averaged across all 21 different BC levels in our data set. The red line shows a linear fit that is forced through zero, for comparison, and the black line is the identity line.

A full description of the algorithm used to generate the correlated responses appears in the Methods section. Here we briefly describe our approach. Correlations in our algorithm are modeled as a doubly stochastic process. The correlations are inserted via hypothetical input variables that are continuous Gaussian random variables with pre-defined uniform (unless otherwise stated) correlation structure; thus, the correlation coefficient between the Gaussian input to cell *i* and the input to a different cell *j* is *c* for all 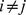. The continuous input variables are then translated to spike-counts via a non-linear transformation, based on matching the cumulative distribution of input variable and the spike-count of the neuron, Figure 3 (see also step 2 of the algorithm in Methods). This transformation retains the marginal spike-count distribution of the neuron. As a result, the input correlations, *c*, generate correlations between the spike-counts, 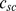, of the neurons via this non-linear transformation. As the transformation from input variables to firing rates is not linear, in general, 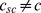. Figure 4 shows the dependence of the firing-rate correlations, 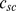, on the input correlation level, *c*, in homogenous populations for different marginal response distributions (in the different panels). The relation between the input correlations, *c*, and the resultant spike count correlations, 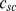, is: monotonic, increasing, with 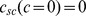 and 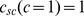, and in many cases can be approximated by: 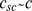. A few cells showed a more considerable deviation from linearity (cells 1&11 Figure 4).

**Figure 5:**
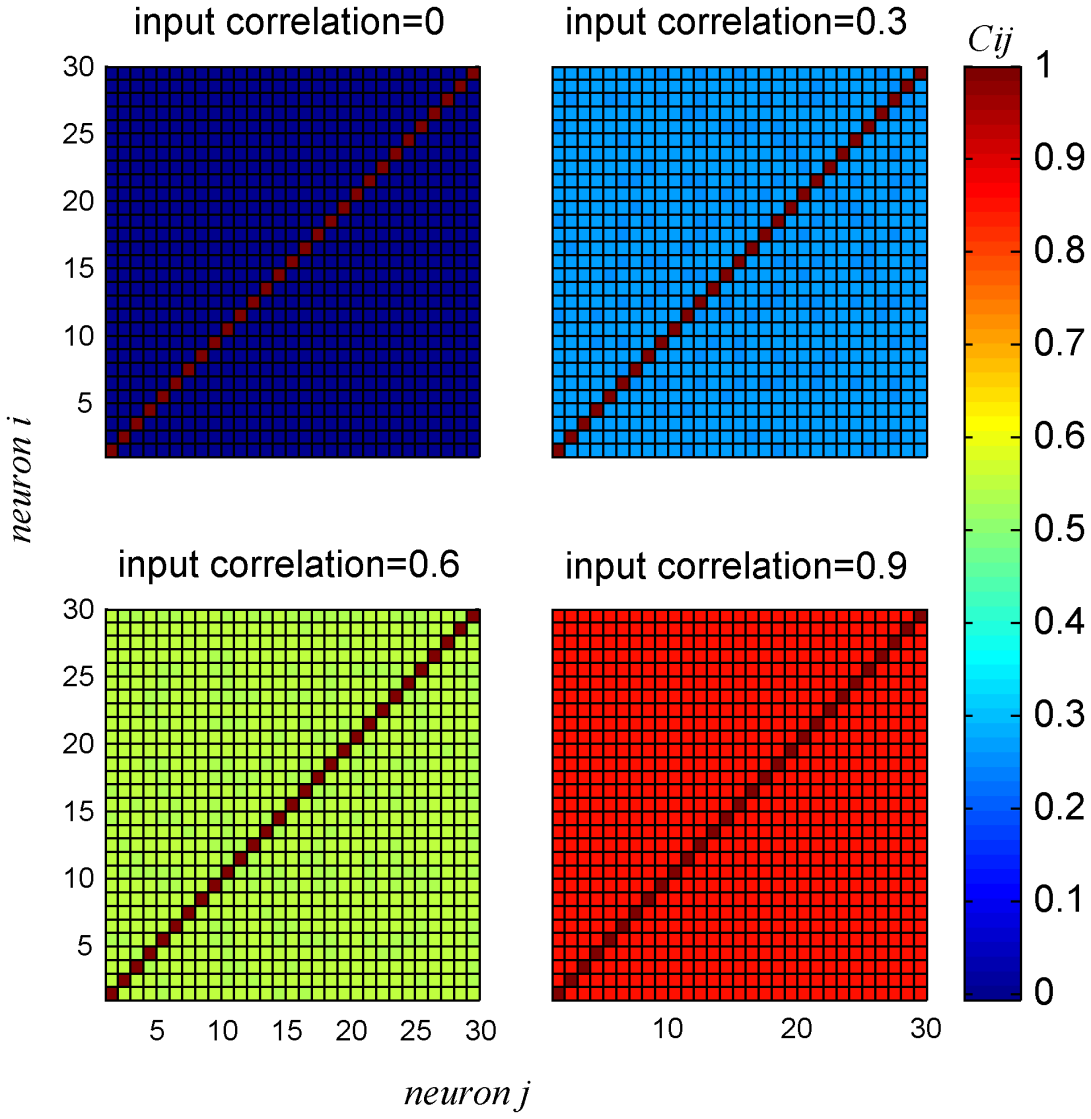
Spike count correlation coefficient 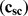 matrices for different levels of input correlation 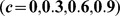 in a homogenous pseudo-population, using the response distribution of cell 9. The correlation coefficients were averaged over all stimulus conditions, 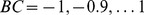. For each stimulus condition the correlation coefficient matrix was estimated by generating 10,000 trials for the pseudo population response for the given stimulus.

Figure 5 shows a typical example of the correlation matrices for the pseudo-population for different levels of input correlations (based on the response of cell 9 of Figure 1). The correlation structure of the neural responses for a homogeneous population is uniform, i.e., the spike-count correlations between every two different neurons in the pseudo-population is the same and deviations result only from the estimation of the spike-count correlations using a finite dataset.

**Figure 6:**
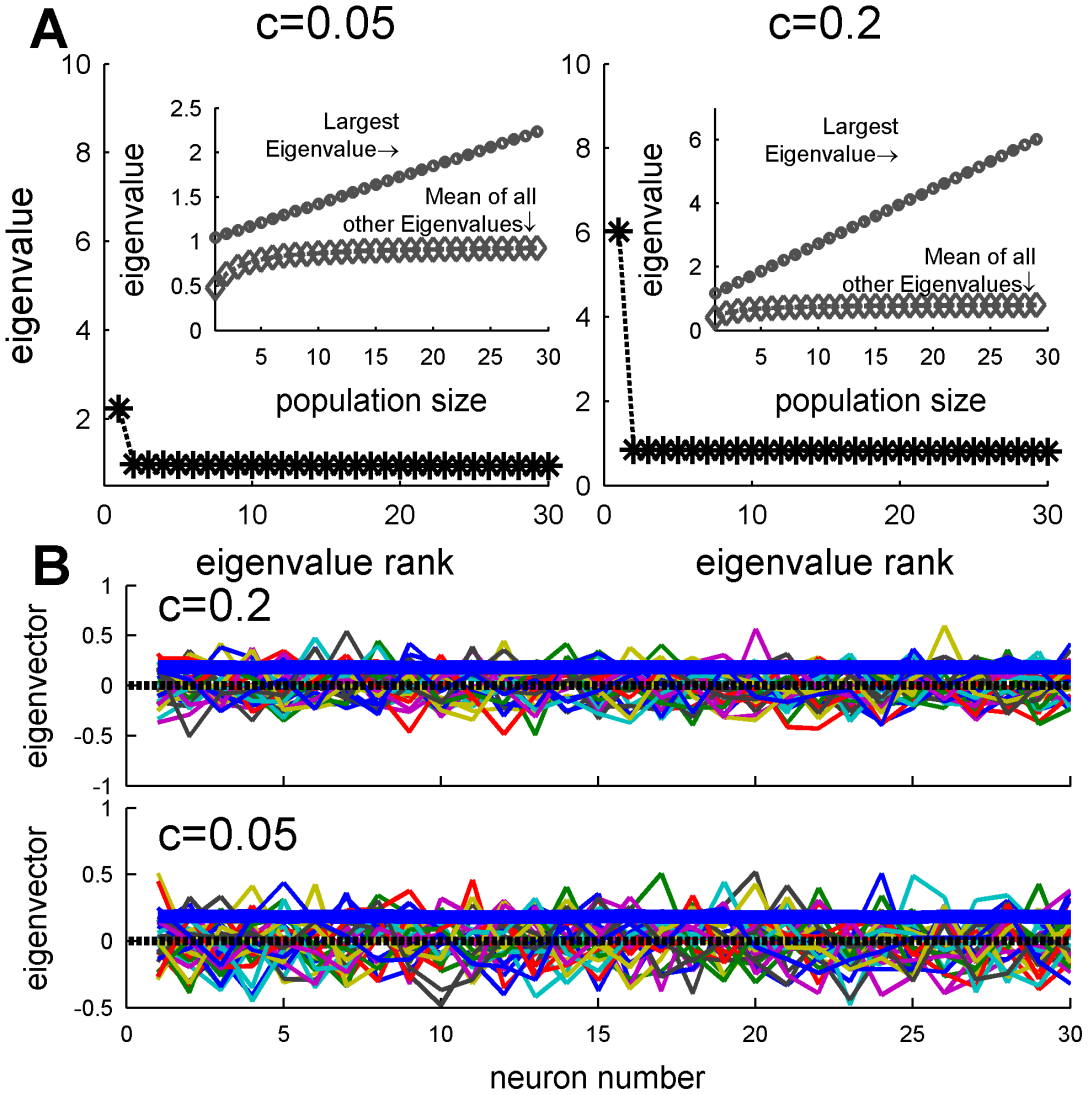
Eigenvalue spectrum and eigenvectors of the spike count correlation coefficient 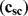 matrix. (A) The spectrum of the correlation matrix of a homogenous pseudo-population of 30 neurons, with input correlations of 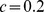, and 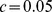 is shown,. The inset shows the largest eigenvalue (circle) and mean of all other eigenvalue (diamond) as function of population size. (B) The eigenvectors of the correlation matrices A are shown by the different colors. The thick blue lines show the eigenvector that corresponds to the largest eigenvalue.

It is useful to analyze the matrix of spike-count correlations into their principle components. These are the groups of cells which fluctuate together, i.e. in a “collective mode”, and which, in statistical analysis, are ordered so that successive components account for decreasing magnitudes of variance. The advantage of this grouping into “collective modes” is that they are uncorrelated with each other (i.e. orthogonal) so are simpler to examine than the mutually correlated neural responses. The structure of each “collective mode” is given by an eigenvector of the correlation matrix, and the magnitude of its fluctuation (variance) is given by the corresponding eigenvalue [27]. The collection of eigenvalues is known as the spectrum of the correlation matrix (e.g. Figure 6A), and allows the magnitude of the different modes to be compared. The eigenvector gives the structure of the mode. For example, a uniform eigenvector, i.e. one in which each component is equal (e.g. solid blue line in Figure 6B), represents a collective mode in which the entire population fluctuates above (or below) its mean firing rate together. Alternatively, one can think of the uniform eigenvector as representing the fluctuations of the “center of mass” of the neural responses.

Figure 6A, shows the eigenvalue spectrum and eigenvectors, respectively, of a correlation matrix with 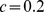 and 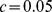. The spectrum of the correlation matrix is composed of one large eigenvalue that grows linearly with the population size (Figure 6A inset) and its corresponding eigenvector (Figure 6B thick blue line) has approximately equal components, i.e. representing a *uniform* mode of fluctuations in which all neurons fire above or below their mean together. The rest of the eigenvectors are degenerate, with values which vary randomly from cell to cell (Figure 6B), and remain on the order of one as the population size grows (Figure 6A inset). Thus, the trial-to-trial fluctuations of the population response are dominated by a collective uniform fluctuation with a magnitude that scales linearly with the population size, whereas the noise in all other orthogonal directions remains of the same order even as the population size increases. In other words this correlation matrix is well modeled by a single principle component. Note, that the correlations do not alter the total noise in the population, as the sum of all eigenvalues equals the sum of all diagonal elements of the correlation matrix, which is equal to the number of neurons in the population. Only the *distribution* of noise is affected by the correlations.

### Linear readout accuracy in correlated homogenous populations

Throughout this paper we will use the accuracy of a simple linear readout as a measure of the information content of the population response. Specifically, we will quantify the accuracy in terms of the reciprocal of the mean square estimation error of the linear readout. The squared estimation error is composed of a sum of two contributions (see Methods). One is the square of the bias, which measures the systematic error and how accurate the estimator is on average. The second is the variance of the estimator that quantifies its trial-to-trial fluctuations. As the bias is deterministic, in many cases, it is ignored. Here, for example, if the tuning curves of the neurons were exactly linear one could obtain an unbiased estimator.

**Figure 7:**
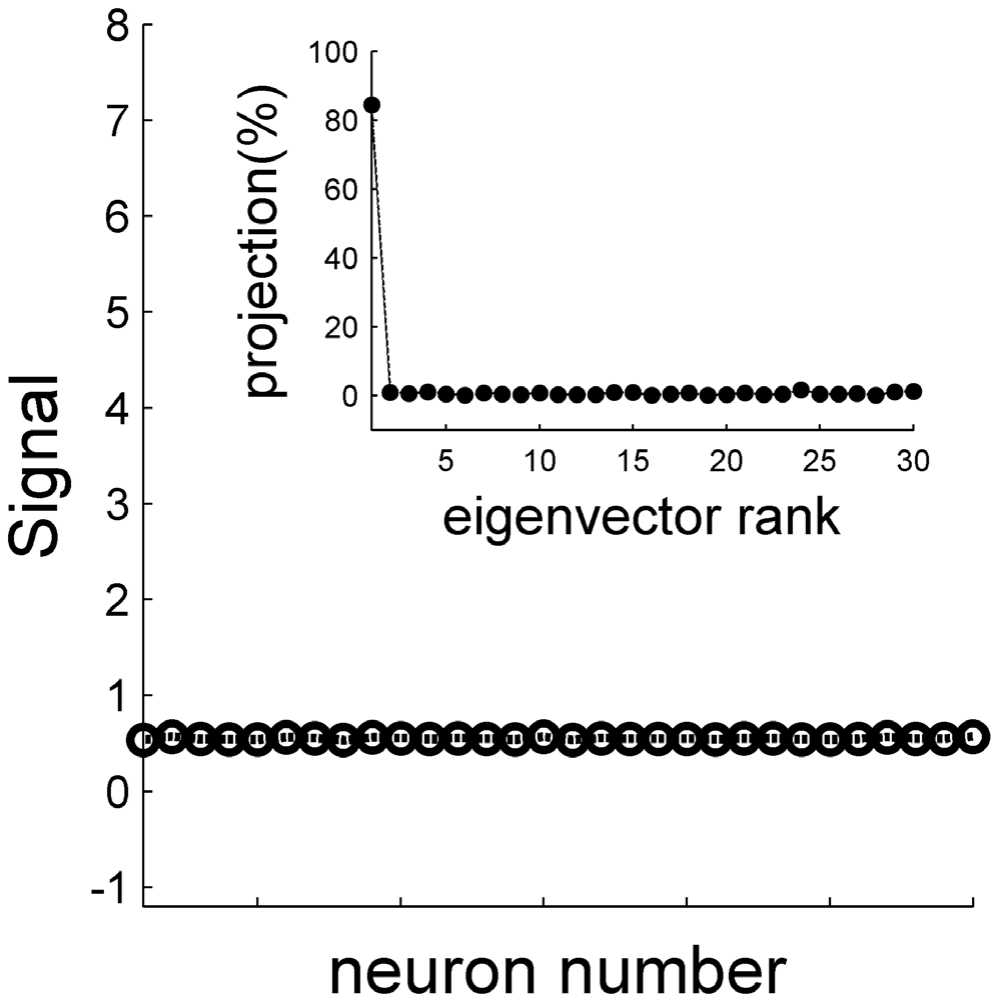
The signal in a homogeneous population. The signal, in terms of the covariance between the neural response and the stimulus is shown as a function of the neuron number in a homogeneous population based on the responses of cell 13. As the population is homogeneous the signal is distributed homogeneously in the responses of the different neurons in the population. The inset shows the distribution of the signal over the different eigenvectors of the neural spike-count correlation matrix (with uniform correlation coefficient of 0.2), as a function of the rank of their eigenvalue. The inset shows the projection of the signal vector on each eigenvalue in percent. Note that the first eigenvector corresponds to the uniform vector (cf Figure 7). The signal and the correlation matrix were estimated using 10,000 repetitions for every stimulus value 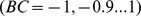.

A linear readout for the BC, 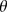, is a linear function of the neural responses 
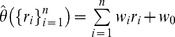
; where 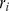 is the response of neuron 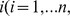 with 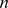 the number of neurons in the population), 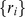 the response set, and 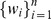 is the set of linear weights and offset, 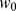. The optimal linear estimator (OLE) is a linear readout with the specific choice of weights that minimizes the mean squared estimation error [28]. The OLE is determined by two factors: noise and signal (see Methods). The noise is quantified by the correlations (analyzed above). The signal measures the *sensitivity* of the neural responses to *changes* in the stimulus, e.g., the tuning curves of the neurons in the population. Specifically, for a linear readout, such as the OLE, the signal is embedded in the covariance of the *mean* neural responses (i.e., tuning curves) with the stimulus. In a homogeneous population, a change in the stimulus will induce the exact same change in the responses of all neurons in the population, Figure 7. Hence, the signal will reside only in the uniform direction, Figure 7 inset.

**Figure 8:**
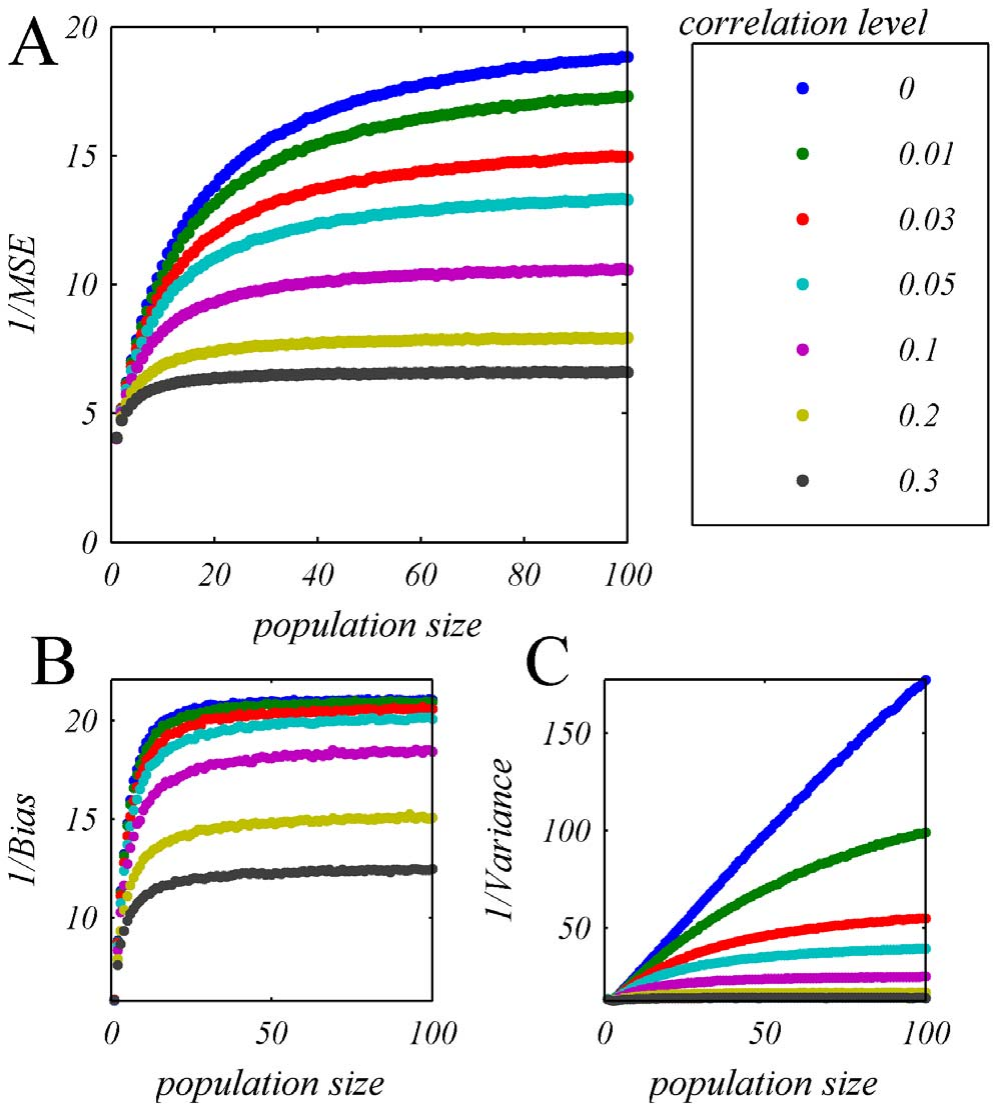
OLE accuracy in a homogeneous pseudo population. The OLE accuracy is shown in terms of: **A** one over the mean square estimation error, and its components: **B** the inverse of the bias and **C** the inverse of the variance, as a function of the number of cells for different levels of uniform correlations, by the different colors. The dots show the estimated OLE accuracy that was measured by first training the OLE weights using 10500 trials of psedo-population response (i.e., 500 trials per stimulus condition) and estimating the accuracy over 10500 trials of generalization, this procedure was repeated and averaged 100 times. Note that as the pseudo-populations are homogeneous and are uniquely determined by the marginal response distribution of a single neuron, there is no need to average over different realizations of the population. Specifically, here we have used the response distribution of cell 13 (Figure 1) to define the population response.

Figure 8 shows the accuracy of the OLE (A) and the contribution of the bias (B) and variance (C) for a typical homogeneous population (defined by the marginal response distribution of cell number 13), as function of population size for different input correlation levels (shown by the different colors) as a function of the population size. The top blue line shows the OLE accuracy in the case of uncorrelated population response. As can be seen from the figure, OLE accuracy saturates even in the absence of correlations. However, examining the two components of the OLE error, i.e., bias and variance, Figure 8B & C, reveals that in the uncorrelated case (blue dots) it is only limited by the bias, which results from the non-linearity of the tuning curves of the neurons and can be easily overcome using a deterministic mapping for large population in the uncorrelated case, 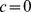. In this case (zero correlations), the variance of the trial-to-trial fluctuations of the OLE decays to zero algebraically in N.

On the other hand, the OLE accuracy, in the correlated case 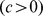, is limited by the variance component, which saturates to a finite value, as does the bias. This trial-to-trial variability cannot be overcome by a deterministic transformation. Thus, even in the presence of low correlation levels the accuracy of the OLE saturates to a finite value. These and similar findings suggested that information content of neural populations is limited due to spike count correlations, which have been empirically observed.

### Correlations in a heterogeneous population

**Figure 9:**
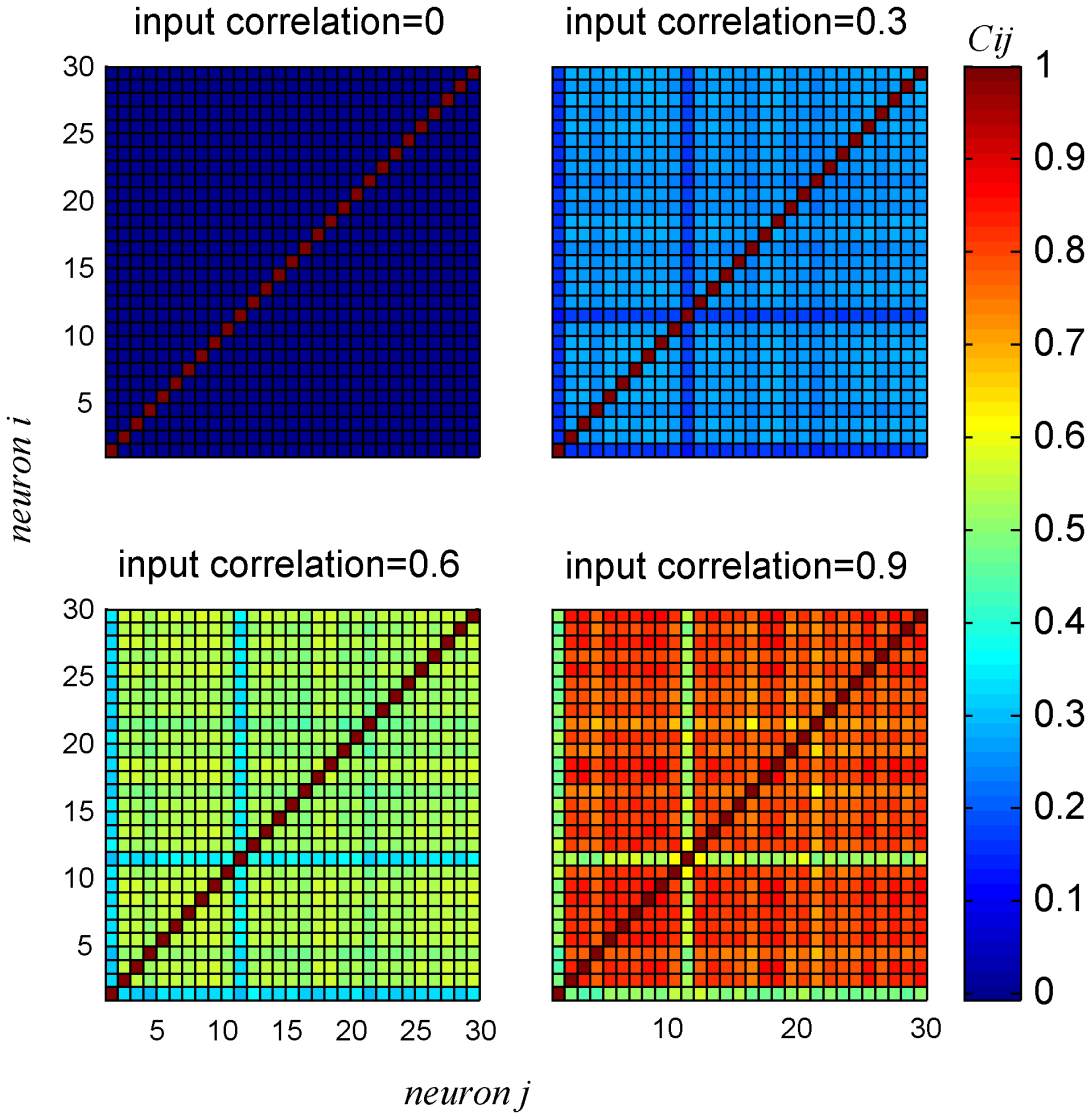
Spike count correlation coefficient 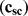 matrices for a specific realization of a heterogeneous population of neurons for different levels of input correlation, 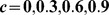 from bottom to top. The correlation coefficients matrix was computed for heterogeneous pseudo-population of 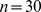 neurons composed of all 30 neurons in the data set. The correlation coefficient matrix was computed by averaging over all stimulus conditions. For each stimulus (BC level), the conditional correlation coefficient matrix was estimated by generating 10,000 realizations of the pseudo population response.

**Figure 10:**
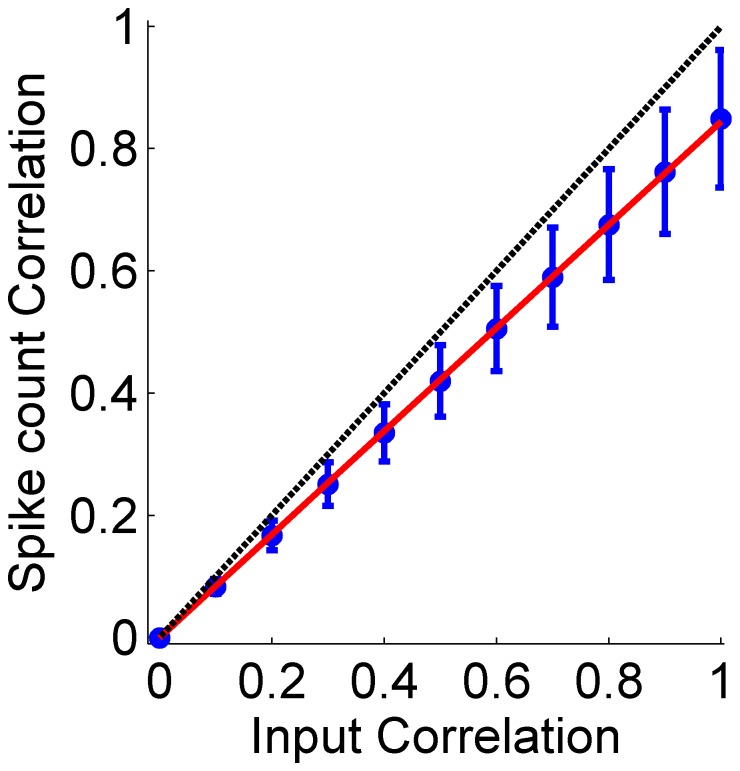
Spike count correlation 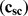 as function of the input correlation strength, 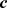, for a heterogeneous pseudo-population. Neural responses for a population of 30 neurons containing all the cells in our data set were generated for all stimulus conditions (i.e., all 21 different BC levels in our data set) and the correlation coefficients for all the different cell pairs were averaged (blue circles), see Methods. The error-bars show the standard deviation of the correlation coefficients in the populations. The solid red line is a linear regression line with slope of 0.85, forced via the origin. For comparison the dashed black line shows the identity line.

Figure 9 shows examples of the resultant correlation matrices in a heterogeneous pseudo-population for four different input correlation levels, as generated by the algorithm. As the transformation of input correlations to spike count correlations depends on the specific details of the response statistics of each neuron, the spike count correlations in a heterogeneous population are not identically uniform (c.f. Figure 5). The relation between the input correlation strength and the resultant mean (across the heterogeneous population cell pairs) spike count correlations is zero for input correlations of zero, monotonic, and approximately linearly increasing, see Figure 10 and compare with Figure 4. Note that for heterogeneous populations we do not expect the spike count correlations to reach one, even for perfectly correlated inputs. This results from the discontinuous mapping of the continuous input variables to the discrete output (spike count) variable of each cell.

**Figure 11:**
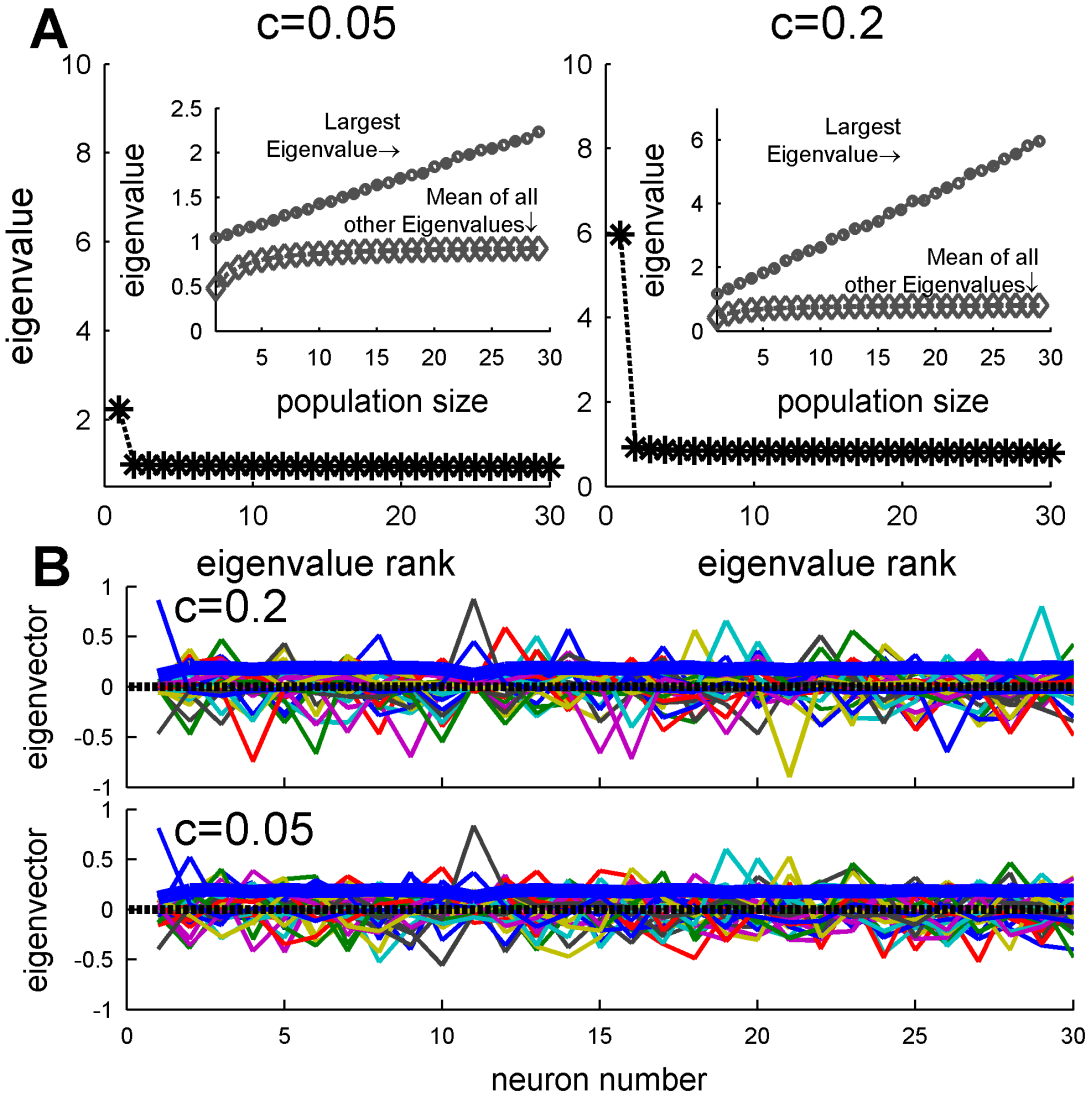
Spectrum and collective modes of fluctuations in a heterogeneous population. (A) Eigenvalue spectrum of the spike count correlation 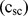 matrix of a heterogeneous pseudo-population containing all 30 neurons in our data set, and input correlations of 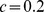 and 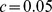. The inset shows the largest eigenvalue (circle) and mean of all other eigenvalues (diamond) as function of population size. (B) The different eigenvectors of the spike-count correlation 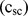 matrix (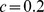 and 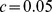) are shown by different colors. The thick blue line depicts the eigenvector with the largest eigenvalue.

Due to the heterogeneity of the population, the correlation structure of the neural responses is not completely flat; compare Figure 5 and Figure 9. Nevertheless, the eigenvalues spectrum and eigenvector structure (Figure 11A) show similar characteristics to the homogeneous case (Figure 6A). As in the homogeneous case, the spectrum of the correlation matrix is composed of a single eigenvalue that grows linearly with the population size, whereas the rest of the eigenvalues reach a size independent limit of order one (Figure 11A inset). Inspection of the eigenvector that corresponds to the largest eigenvalue (Figure 11B thick blue line) reveals a vector with nearly constant coefficients, i.e. it has a high overlap with the uniform vector. Thus, although the correlation matrix in the heterogeneous case is not completely flat, its structure remains the same. The noise in the population response is composed of a single collective mode of fluctuation (the principal component) that is relatively uniform with a magnitude that increases linearly with population size whereas the magnitude of the rest of the eigenvalues do not scale with the population size.

### Information content of a heterogeneous population

**Figure 12:**
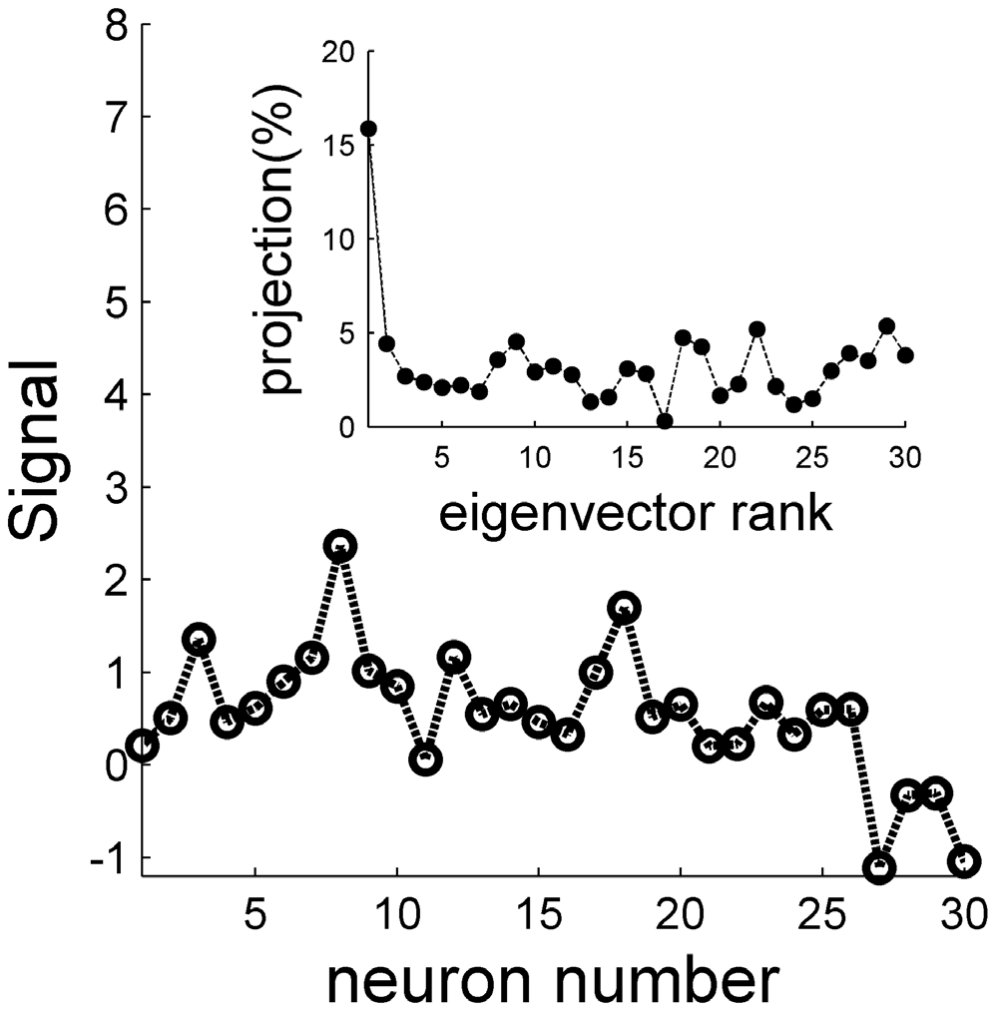
The signal in a heterogeneous population. The signal, in terms of the covariance between the neural response and the stimulus is shown as a function of the cell number in a heterogeneous population based on the responses of all cells in the data set. The inset shows the distribution of the signal over the different eigenvectors of the neural spike-count correlation matrix (with uniform correlation coefficient of 0.2), as a function of the rank of their eigenvalue, in percent. Note that the first eigenvector corresponds to the uniform vector (cf Figure 13). The signal and the correlation matrix were estimated using 10,000 repetitions for every stimulus value 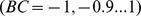.

We next studied the information content of the neural responses in a heterogeneous population. In contrast with the noise that is very similar to the homogeneous case, the signal is qualitatively different, Figure 12. Due to the heterogeneity, the signal is no longer uniform. Figure 12 inset shows the distribution of the signal over the different modes (eigenvectors) of the correlation matrix. Although the signal distribution peaks at the uniform direction (the principle component), only about 15% of the signal resides in that mode. Hence, a considerable portion of the signal resides in modes in which the noise does not increase with the population size.

**Figure 13:**
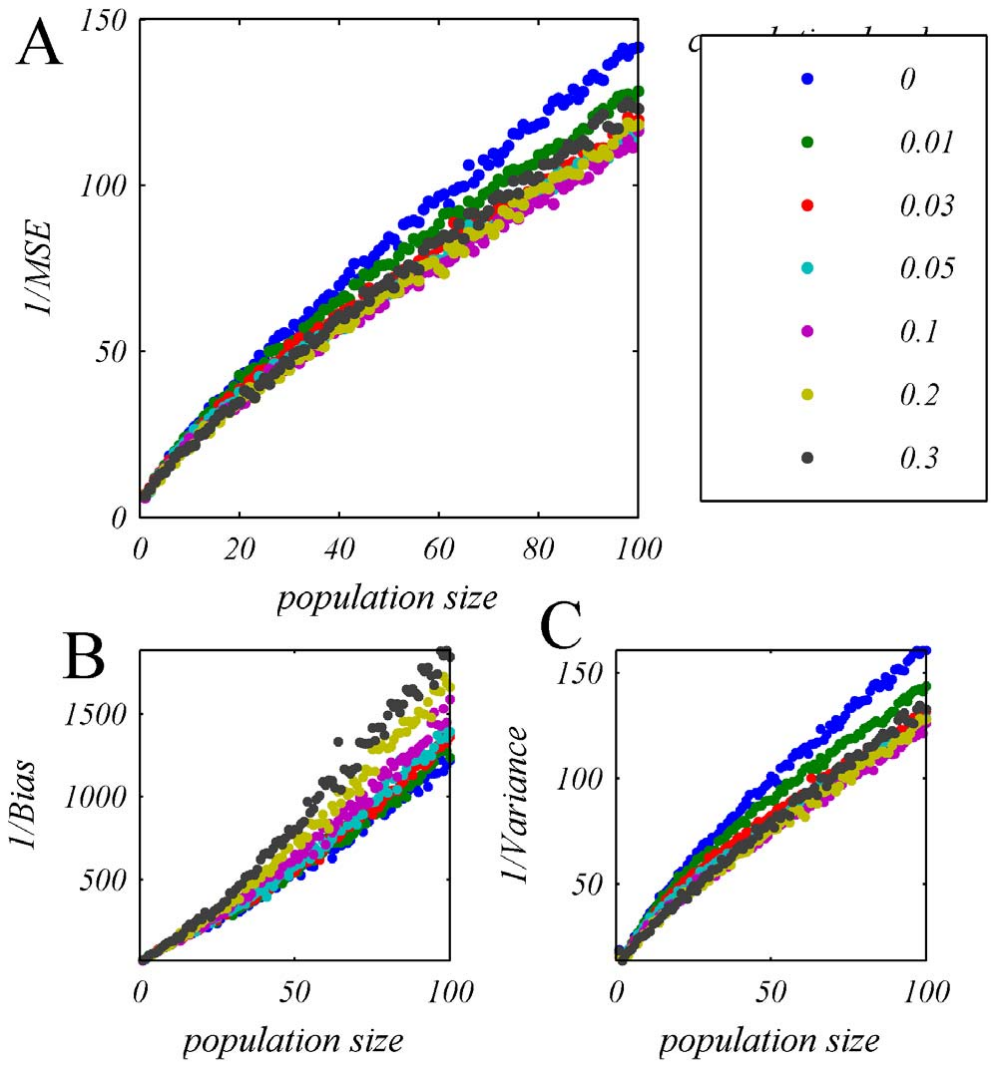
One over the Mean Square Error (A), bias (B) and variance (C) of OLE estimation, plotted as function of population size for heterogeneous pseudo-population with different input correlation level (different color). All heterogeneous pseudo-populations created based on empirical response statistics of 30 cells. The OLE weights were learned from a training set of 500 trials per stimulus condition and the accuracy of the readout was estimated using a generalization set of the same size. For each value of population size the accuracy was averaged over 100 realizations of the neural population, where the different cells in each realization were drawn independently with equal probabilities (with repetitions) from the pool of all neurons.

The accuracy of the OLE, in terms of the reciprocal of the mean squared estimation error and its components, i.e., the reciprocal of the bias and the reciprocal of the variance, are shown as a function of the population size for different levels of mean correlation, Figure 13. In contrast with the case of homogenous population (Figure 8), the OLE accuracy increases with the population size, and the two components of the OLE accuracy (i.e., bias and variance, Figure 13 B and C, respectively), scale approximately linearly with population size even at high levels of correlation. Thus, even in the presence of high correlation levels the accuracy of the OLE does not saturate to a finite value and keeps growing linearly with population size. These and similar findings suggested that for a linear estimator that is fine-tuned to the heterogeneity of a population, information content is not limited by the empirically observed spike count correlations.

**Figure 14:**
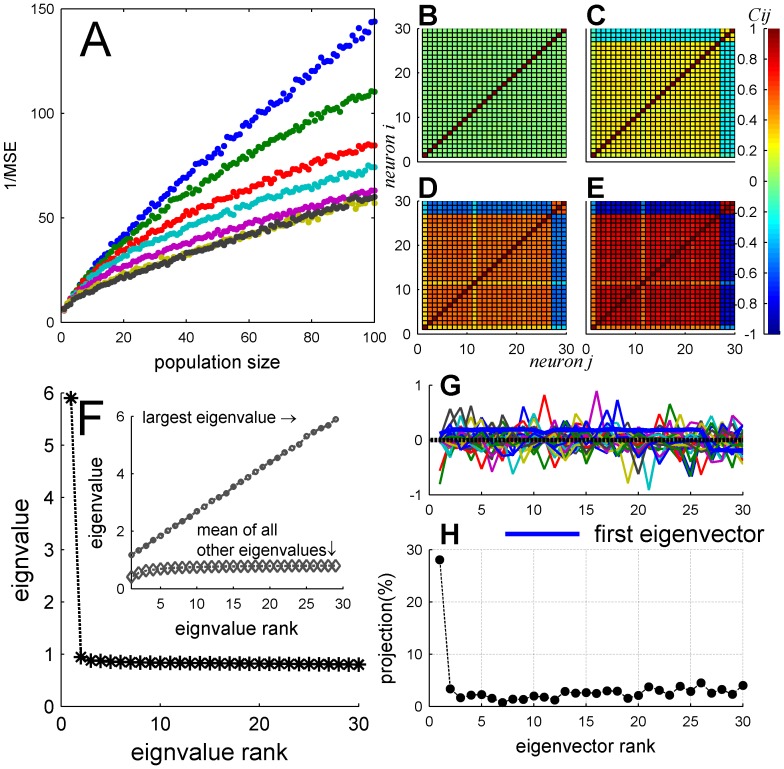
Functionally dependent correlations. (A) One over the Mean Square Error of OLE estimation, plotted as function of population size for heterogeneous pseudo-population with different *functionally dependent* input correlation level (different color). All heterogeneous pseudo-populations created based on empirical response statistics of 30 cells. The OLE weights were learned from a training set of 500 trials per stimulus condition and the accuracy of the readout was estimated using a generalization set of the same size. For each value of population size the accuracy was averaged over 100 realizations of the neural population, where the different cells in each realization were drawn independently with equal probabilities (with repetitions) from the pool of all neurons. (B–E) Four examples of the neuronal noise correlation matrix (in color code) for different values of functionally dependent input correlations, 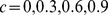 respectively. The matrices were estimated by averaging the conditional correlation coefficient matrices [estimated using 10,000 trials] over all stimulus conditions. (F) Eigenvalue spectrum of the functionally dependent correlation matrix of a heterogeneous pseudo-population containing all 30 neurons in our data set, and functionally dependent input correlations of 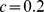. The inset shows the largest eigenvalue (circle) and mean of all other eigenvalues (diamond) as function of population size. (G) The eigenvectors plotted from output correlation 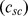 matrix 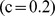 of the functionally dependent correlation matrix. Each vector plotted in different color. The thick blue line shows the eigenvector with the largest eigenvalues. (H) The signal distribution across the eigenvectors of the spike-count correlation matrix is shown as a function of the eigenvalue rank.

Empirical studies reported that in many cases noise-correlations are functionally-dependent. This functional dependence gives rise to collective modes of fluctuations that look like the signal. In a homogeneous population coding for BC there is only one preferred stimulus, and as a result functionally-dependent correlations are homogeneous correlations. Neurons in our data set can be thought of as originating from two distinct columns. One column with preferred BC of 1 (about 85% of the neurons), and the other with preferred BC of −1 (about 15%). We tested the effect of functionally-dependent correlations on the accuracy of the OLE in a heterogeneous population, Figure 14 A (see also Methods). Figures 14 B–E show the spike-count correlation matrix for varying levels of functionally-dependent correlations. Note the similarity between the noise correlation of e.g. Figure 14 E and signal correlations, Figure 2. The noise in the neuronal response, in this example, is composed of a single collective mode that grows linearly with the population size, Figure 14 F, and the structure of the largest collective mode respects the functional distance between the neurons, Figure 14 G. The distribution of the signal across the different eigenvectors of spike-count correlation matrix is shown in Figure 14 H. The mode (in the meaning of the most common value in the distribution) of the signal distribution is in the principle component with 30%, compare with 15% in the case of uniform correlations Figure 12. However, due to the heterogeneity of the neural responses 70% of the signal is distributed in directions in which the spike-count correlations do not grow with the population size. Thus, although the effect of correlations is larger than in the case of uniform correlations (compare with Figure 13) the distribution of the signal enables the accuracy of the OLE to grow linearly with the population size.

## Discussion

There is abundant evidence for neural response correlations in the central nervous system [7], [13], [21], [29]–[32]. However, only few studies have investigated spike count correlations between different neurons in the IC, yet there are findings suggesting that the responses of different cells in the auditory system and in particular in the IC display correlation [5], [33]. Correlation values in many cortical areas are found over a relatively broad range, from 0.02 to 0.26, although the value of correlation seems to be sensitive to the methods of measurement [7], [10].

Here, we used the coding of BC by IC neurons as a convenient framework to study the effect of correlated noise and neuronal heterogeneity on the ability to accumulate information from large populations of neurons. The BC of the stimulus is represented by a continuous parameter with values in the range from minus one to one, and is characterized with tuning curves that are relatively linear.

We find that uniform correlations in a homogeneous population limit the information content of the neural responses. The reason is that since all cells are identical they encode information about the stimulus in exactly the same manner. Thus only the mode in which all cells move together, i.e. are equally weighted, gives information about BC. Said differently, due to symmetry the only direction in the phase space of the neural responses of a homogenous population that encodes information about the stimulus is the uniform direction, i.e., only the ‘center of mass’ of the neural responses carries information about the BC. However, due to the correlations, the noise in the uniform direction grows linearly with the population size (see inset of Figure 6A), i.e., the ‘center of mass’ of the neural responses is the principle component of the noise fluctuations with a variance that grows linearly with the population size. Other directions do not encode information that can be “read out” by linear readout mechanisms.

**Figure 15:**
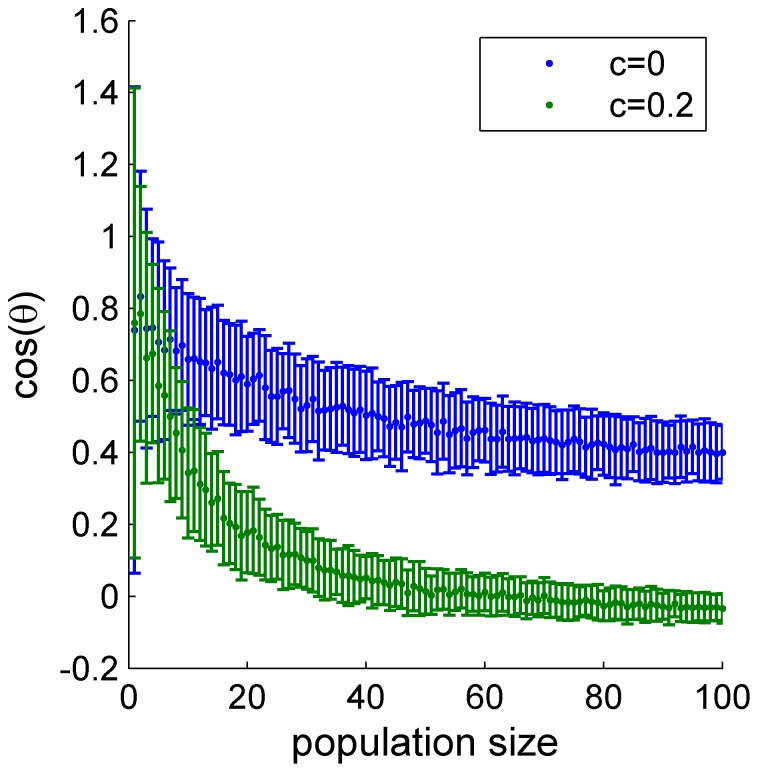
The overlap between the OLE weights and the uniform direction. The cosine of the angle between the OLE weights vector and uniform vector is plotted as function of population size without correlations (blue) and with input correlation of 0.2 (green), in a heterogeneous population.

In contrast, in a heterogeneous population the accuracy of the OLE grows linearly with the population size, even in the presence of correlations. Comparing Figures 6 and 11, it is clear that the noise structure is similar in both cases. Hence, the ability of the OLE to overcome the correlated noise in the heterogeneous case results from the different distribution of the signal, i.e., the modes of the network that are sensitive to the stimulus. Since the heterogeneity information on the BC is not limited to the uniform direction, and, as a result, information about the stimulus can be extracted also from non-uniform directions of the neural response, in which the noise remains finite (Figure 11A inset). This is illustrated in Figure 15 that shows the relative weight of the ‘center of mass’ (uniform direction) in the OLE, as a function of the number of cells in the population. In an uncorrelated population, 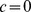, (blue) there is a considerable contribution to the optimal weights from the center of mass, as the center of mass contains most, but not all, of the signal. On the other hand, in the presence of uniform correlations, the noise in the center of mass grows with the population size (Figure 11). As a result, the contribution of the center of mass to the OLE decreases as the number of neurons increase (Figure 15, green).

We, and others, reported uniform correlations *increase* coding accuracy, whereas ‘limited range’ correlations have been reported to have detrimental effect on coding accuracy [11], [15], [34]. How can these seemingly contradicting finding be explained?

Previous studies focused on the coding of an angular stimulus-variable by a hypercolumn population of neurons with preferred stimuli that are evenly distributed on the ring from −180° to +180°. The term homogeneous has been used loosely in the context of a hypercolumn model to mean neurons which have different preferred stimuli, but all other parameters (e.g. tuning width and maximum firing rate) are the same. We would term this property isotropy, as clearly neurons with different preferred stimuli respond differently. Due to the identical parameters and the uniform distribution of preferred stimuli on the ring, there is no signal in the uniform mode. Adding correlations does not add noise, as the total noise in the system is fixed – it only re-distributes the noise. Thus, adding uniform correlations in an isotropic hypercolumn channels the noise to a mode that has no signal in it. As a result less noise will remain in other modes of the system; hence, the accuracy will increase [11].

We define long range correlations to be such that every neuron is strongly correlated with a fraction of the entire population; hence, generate collective modes of fluctuations. The term ‘limited range’ has been used in the context of a hypercolumn model to describe correlations that are stronger for pairs of neurons with closer preferred orientations. As limited range correlations are (or may be) also long range, a better terminology would be ‘functionally dependent’, that is correlations that depend on the functional distance between neurons (e.g., the difference in their preferred stimuli). When long range correlations are also functionally dependent the collective modes overlap the region where the signal resides in an isotropic hypercolumn; hence, will yield finite signal to noise ratio and a saturation of the accuracy [11], [18].

The noise-correlations in this study are artificial. As we do not have simultaneous recordings we cannot determine their true structure. Thus, the correlations in this work reflect our choice of structure and not necessarily a “real” one. On the other hand, this choice allowed us to focus on the type of correlations that, in the absence of heterogeneity, are the most detrimental ones in terms of coding accuracy. This raises an interesting question regarding the correlations structure and its effect on information capacity of the network: Can correlations increase the information capacity of the system, and if so then what are the optimal correlations?

The trivial answer to the above question is that optimal correlations, in terms of information content, are correlations with zero variance. The smaller the noise the larger the signal-to-noise ratio is. However, this solution is not biological, as we know neural responses are variable. Thus, our empirical knowledge of biology indicates it is not optimal. Nevertheless, it is still tempting to ask: Given the single cell variability, what is the optimal cross correlation structure?

**Figure 16:**
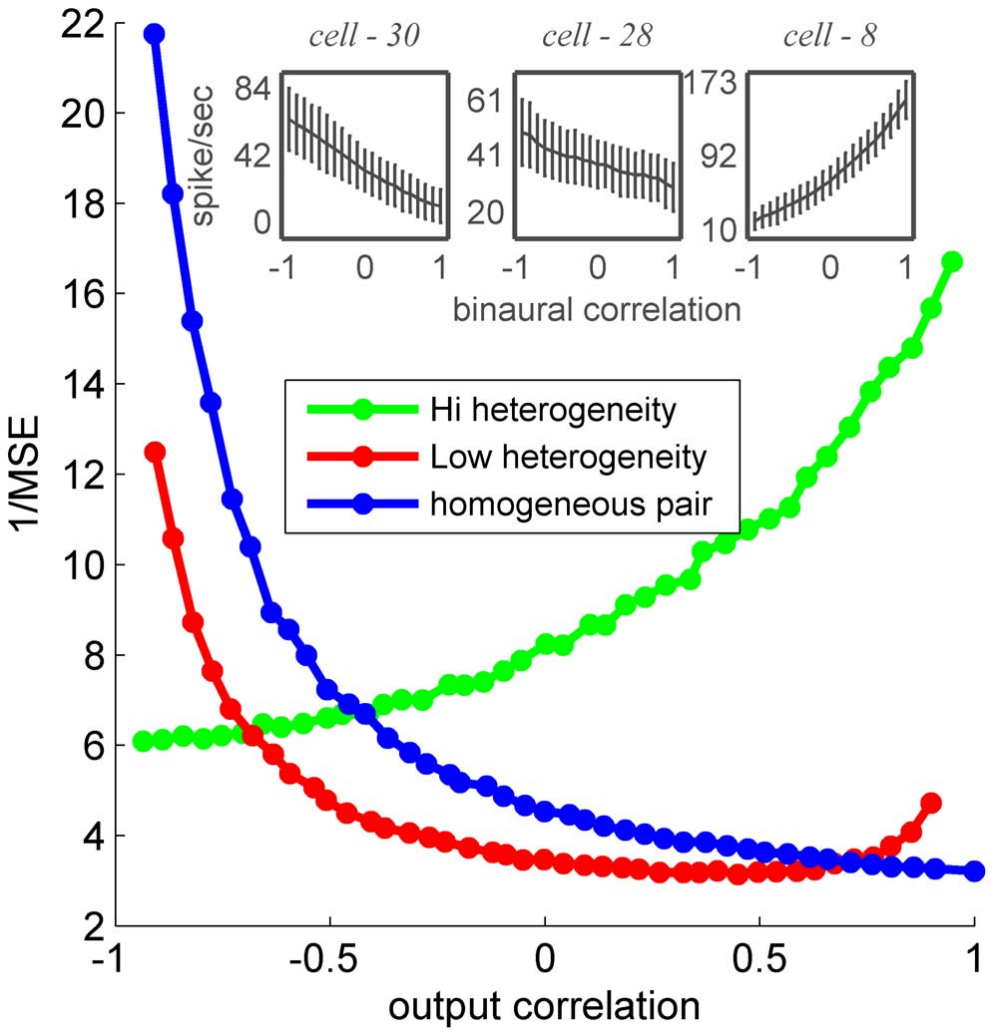
MSE of estimation made by an OLE based on pseudo-population of two neurons plotted as function of spike-count correlation 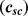 Low heterogeneity pair (red) two cells generated using cells 28 & 30 response statistics, both tuning curves has slops that are monotonic and with the same sign. Homogenous pair (blue) both cells response generated using cell 30 response statistics (cells tuning curves are identical). High heterogeneity pair (green) response generated based on the response of cells 30 & 8.

This question was examined in the framework of a population of two neurons. As the variances are fixed, the entire correlation structure is determined by a single parameter, the spike-count correlation. Figure 16 shows the accuracy of an OLE as a function of the spike-count correlation between the two cells, for three pairs of neurons: a homogeneous pair (blue), a pair with low heterogeneity, i.e., high signal correlations, (red) and a pair with high heterogeneity (green). In a two dimensional system, the trial-to-trial fluctuations (normalized by their standard deviations) can be factored into two collective modes or principle components. One mode is the uniform mode, i.e., the center of mass, in which the two neurons fluctuate together, and the orthogonal mode is the relative mode, in which the two neurons fluctuate with opposite signs (i.e., when one fires above its mean the other fires below). As the variances are fixed, the sum of the strengths of the fluctuations in both modes remains constant. When the correlation coefficient increases, the strength of the uniform mode of fluctuations increases, and as a result, the strength of the fluctuations in the relative mode decreases (improving the SNR on that dimension).

In the homogeneous population (blue), information is embedded only in the uniform direction, i.e., the ‘center of mass’; hence, the OLE accuracy is a monotonically decreasing function of the spike-count correlation. In the heterogeneous population with positive signal correlations, most of the signal resides in the uniform direction. As the spike-count correlation approaches minus one, the noise in the uniform mode decreases to zero and the signal to noise ratio increases, so accuracy increases. In contrast with the homogeneous case, in a heterogeneous population there is also some signal in the orthogonal, relative, mode. For the pair with positive signal correlations (red), the signal in the orthogonal mode is small, but, as the spike-count correlation approaches one, the noise in that mode decreases to zero, yielding increasing signal-to-noise ratio. Thus, the seemingly paradoxical result that increasing spike-count correlation results in an increase in accuracy is due to structure of the noise-correlation matrix. Similar behavior can be observed in the heterogeneous population with negative signal correlations (green) with the difference that in the latter case there is more signal in the orthogonal direction than in the uniform. Thus, we have reached the same trivial answer albeit in a more sophisticated manner: the optimal correlation structure is one that has zero variance in some direction with non-zero signal. Note that this basic explanation using the distribution of noise and signal in the system accounts for the finding that OLE accuracy in a heterogeneous population is not necessarily monotonic in the correlation strength (Figure 16, red curve). Nevertheless, we emphasize that the question of the strength and structure of neuronal noise correlations is not a philosophical one, but rather an empirical one that should be addressed by careful measurement. With respect to the relation of correlation structure and readout accuracy, it is interesting to note that attention and learning have been reported to have a significant effect on the noise-correlations and their structure [8], [35]–[37].

Throughout this work we focused on the information content of the neural response in terms of the accuracy of linear readout mechanisms. A more general readout mechanism may yield a more accurate estimate of the external stimulus [38], [39]. Nevertheless, the computational principles demonstrated here using the framework of linear readout also hold in general. Thus, although correlations are measured throughout the brain, we believe that the inherent neuronal heterogeneity is sufficient to overcome their detrimental effect. On a broader perspective the theory of population coding has focused on quantifying the accuracy of specific hypotheses for the neural readout mechanism. The utility of that approach has been in its ability to reject certain hypotheses that cannot account for the behavioral accuracy. However, this approach cannot assist in discriminating between different possible mechanisms with sufficient accuracy. New theoretical approaches for the study of the neural code are now required.

## Materials and Methods

### Ethics statement

The experiments described in this study were performed under the terms and conditions of licences issued by the UK Home Office under the Animals [Scientific Procedures] Act 1986, project licence number 4003049, and the approval of the ethical review committee of the University of Nottingham.

*Experimental*. The experimental procedure has been described in detail previously (Shackleton et al 2005). In short, recordings were made in the right IC of 7 pigmented guinea pigs weighing 342–779 grams. Animals were anaesthetized with urethane (1.3 g/kg i.p., in 20% solution in 0.9% saline) and Hypnorm (Janssen; 0.2 ml i.m., comprising fentanyl citrate 0.315 mg/ml and fluanisone 10 mg/ml). The animals were placed inside a sound attenuating room in a stereotaxic frame in which hollow plastic speculae replaced the ear bars to allow sound presentation and direct visualization of the tympanic membrane. A craniotomy was performed over the position of the IC. All experiments were carried out in accordance with the UK Animal (Scientific Procedures) Act of 1986. Recordings were made from single, well-isolated neurons, with glass-insulated tungsten electrodes (Bullock et al. 1988) advanced into the inferior colliculus through the intact cerebral cortex, in a vertical penetration. Extracellular action potentials were amplified (Axoprobe 1A; Axon Instruments, Foster City, CA, USA), filtered between 300 Hz and 2 kHz, discriminated using a level-crossing detector (SD1; Tucker-Davies Technologies, Alachua, FL), and their time of occurrence was recorded with a resolution of 1 µs. Stimuli were delivered to each ear through sealed acoustic systems comprising custom-modified Realistic 40–1377 tweeters (M. Ravicz, Eaton Peabody Laboratory, Boston, MA,USA). All stimuli were digitally synthesized (System II, Tucker-Davies Technologies) at between 100 and 200 kHz sampling rates and were output through a waveform reconstruction filter set at one fourth the sampling rate (135 dB/octave elliptic: Kemo 1608/500/01 modules supported by custom electronics). Signals were of 50-ms duration and were presented at 20 dB above the uncorrelated noise threshold. A single repeat consisted of the full range of interaural correlation steps presented in pseudorandom order. Interaural correlation was controlled using the well-known “two-independent noise generator” method (Jeffress and Robinson 1962). Briefly, two independent noise samples were generated. One of these was presented to the left ear. The signal presented to the right ear was a sum of that presented to the left ear, and the other independent noise in the proportion 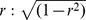 (e.g., Culling et al. 2001, Eq. A1).

*Data analysis*. The conditional mean firing rate of each neuron, as a function of stimulus BC was estimated from the data by averaging the spike count during 100 ms following stimulus onset, over all 200–500 trials for each of the 21 different stimuli 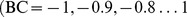: Figure 1). Note the error bars on Figure 1 give the standard deviation of the neuron's response and hence indicate the variability in spike count. To obtain the standard error (standard deviation of the estimate of the mean) one has to divide by the square root of the numbers of trials per stimulus; hence there is a factor of about 15–20 between the shown standard deviation and the standard error of the mean. The entire data set was used for the empirical estimation of the neural cumulative response probability. This distribution was used to generate the pseudo-population response (see below). As the correlation coefficients of the responses of different neurons are not independent of the stimulus, in all presented figure we show the average correlation coefficient over the 21 stimulus conditions.

Information content of the neural response was measured in terms of the reciprocal of the mean square estimation error. The optimal linear estimator (OLE, [28]) is defined by the specific choice of linear weights that minimizes the mean square estimation error,
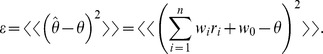
where 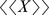 denotes averaging of 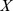 with respect to the stimulus and the neural distribution. The mean square error can be written as the sum of two components: the (squared) bias and the variance of the estimator, 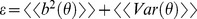, where the bias is the conditional mean of the estimator for a given stimulus. The bias and variance that are shown throughout the paper are averages of the squared bias and the variance over all stimulus conditions.

In the case of a heterogeneous population the identity of the cells in the population was chosen randomly from the pool of neurons. The cells were drawn from the pool independently with equal probabilities and with repetitions. For every population size, the accuracy of the estimator was averaged over 100 realizations of the neural population

### Generating a homogeneous pseudo-population response

The following procedure generates the response of a statistically independent homogeneous population of neurons. The procedure is equivalent to creating a pseudo-population of cells by independently sampling the responses of the same neuron repeatedly with replacement. The utility of this formalism is that it is easier to generalize it in order to implement trial-to-trial correlations. To simulate the response of a pseudo-population of *n* cells in response to auditory stimulus with BC level of 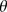 based on single cell data of a specific cell 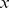 we applied the following two-step algorithm:

Step 1: for every cell, 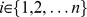, in the pseudo-population we generated random variables 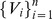. The variables 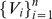 were independent and identically normal Gaussian distributed
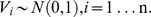
The variable 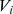 can be thought of as the input to cell 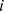 or, alternatively, as its underlying membrane potential.

Step 2: To translate the input variables 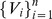 to spike count 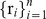 while preserving the *marginal* distribution of the single neuron 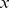 response statistics for a given BC level of 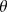 we used the following procedure, see Figure 3. Denote by 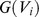 the cumulative distribution of the input variable 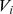, 
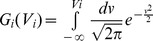
, and by 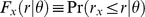 the cumulative probability that neuron 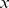 of the dataset will fire *r* spikes or less in response to stimulus 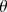. The spike count of neuron 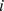 in the pseudo population is set to be 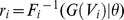. Note that as the spike count 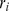 is a discrete variable we choose 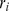 such that 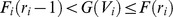.

### Generating a pseudo-population with spike count correlations

To model correlations between neurons we modify step 1 of the algorithm. Correlations in our algorithm are inserted via the inputs, 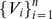. In a correlated population, each input 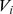 is composed of a sum:
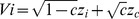
Where 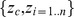 are independent and identically distributed normal Gaussian random variables:
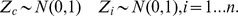
The term 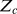 denotes the shared input that is common for all cells. The term 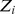 is the independent component of the input to cell *i*. The parameter *c* determines the correlation coefficient between the *inputs* to the different cells in the population. Thus the set of inputs are random Gaussian variables with zero mean, variance of one and uniform correlation coefficient, 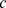.

To generate functionally dependent correlations we inverted the sign of the shared\ correlated component of the input variable to neurons with negative slope tuning curves (neurons 27–30 in Figure 1). The input to neuron *i* with positive slope was 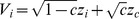, and to neuron *j* with negative slope 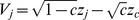. Thus the collective mode of fluctuations resembles the vector 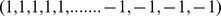.

### Generating a heterogeneous pseudo-population

To generate a heterogeneous pseudo-population we simply used the cumulative spike-count distributions of different cells in step two of the algorithm. The different cumulative spiking distributions 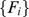, were chosen randomly with equal probabilities with repetitions in an independent manner. Cell responses in the pseudo-population preserve the marginal response distributions of cells from which they were generated by the algorithm; thus creating a heterogeneous population.
